# Mitochondrial DNA Abnormalities in Idiopathic Inflammatory Myopathies: Innate Immune Signaling and Therapeutic Prospects

**DOI:** 10.3390/ijms27219949

**Published:** 2026-11-06

**Authors:** Guanyuan Wang, Yurui Wang, Xinran Wang, Karl J. A. McCullagh, Aijing Liu

**Affiliations:** 1 Department of Rheumatology and Immunology, The Second Hospital of Hebei Medical University, Shijiazhuang 050000, China; guanyuanwanghmu@163.com (G.W.); wyr202403@126.com (Y.W.); xinranwanghmu@163.com (X.W.); 2 Hebei Medical University-National University of Ireland Galway Stem Cell Research Center, Hebei Medical University, Shijiazhuang 050017, China; 3 Hebei International Joint Research Center on Rheumatic Diseases, Shijiazhuang 050000, China; 4 Discipline of Physiology, School of Pharmacy & Medical Sciences, Human Biology Building, University of Galway, H91 W5P7 Galway, Ireland; 5 Hebei Research Center for Stem Cell Medical Translational Engineering, Shijiazhuang 050017, China

**Keywords:** idiopathic inflammatory myopathies, mitochondrial DNA, cGAS–STING pathway, TLR9, NLRP3 inflammasome, biomarkers, therapeutic strategies

## Abstract

Mitochondrial DNA (mtDNA) encodes essential components of oxidative phosphorylation and can stimulate innate immunity when released from mitochondria. Its genetic and immunological functions provide a rationale for investigating mtDNA abnormalities in idiopathic inflammatory myopathies (IIMs), where muscle dysfunction and inflammation coexist. Large-scale deletions and depletion have been documented in inclusion body myositis and polymyositis with mitochondrial pathology, while dermatomyositis studies reveal regional depletion and oxidative modifications in distinct biological compartments. These findings raise the possibility that mtDNA abnormalities contribute to both impaired mitochondrial function and inflammatory signaling. This review integrates subtype-specific findings with mechanistic studies of mtDNA release, DNA sensing, and inflammasome activation in IIM-related and non-IIM systems. It evaluates the evidence for mtDNA-based biomarkers and therapies targeting mitochondrial injury or downstream inflammation. The reported clinical benefits do not establish mtDNA-specific therapeutic effects. Defining how mitochondrial genome abnormalities and inflammatory signaling interact within each subtype is a priority for biomarker validation and therapeutic research.

## 1. Introduction

Idiopathic inflammatory myopathies (IIMs) comprise a heterogeneous family of systemic autoimmune diseases driven primarily by chronic skeletal muscle inflammation and immune-mediated myofiber injury. According to distinct clinical manifestations, histopathological features, and myositis-specific autoantibody (MSA) profiles, IIMs are mainly subclassified into dermatomyositis (DM), immune-mediated necrotizing myopathy (IMNM), antisynthetase syndrome (ASyS), sporadic inclusion body myositis (sIBM), and overlap myositis [1,2]. Globally, the incidence of IIMs ranges from 0.2 to 2 per 100,000 person-years, with prevalence estimates of 2–25 cases per 100,000 people [3]. Although progressive proximal muscle weakness, reduced endurance, and myalgia constitute the cardinal presenting manifestations, extramuscular involvement affecting the skin, joints, lungs, heart, and other organs frequently complicates the disease course. At present, the standard-of-care treatment of IIMs other than sIBM chiefly relies on glucocorticoids combined with conventional immunosuppressants and achieves remission in some patients [1,4]. However, responses remain unpredictable, and relapse, treatment resistance, or persistent functional disability occur in a substantial proportion of patients and adversely affect long-term quality of life [1,5]. Long-term glucocorticoid exposure also carries risks, including osteoporosis and osteonecrosis [6,7].

The clinical diagnosis and subtype classification of IIMs are based on an integrated assessment of muscle weakness patterns, extramuscular manifestations, serological findings, and muscle histopathology. DM is supported by characteristic skin lesions and perifascicular pathology, with MSAs defining clinically relevant subsets. Anti-signal recognition particle (anti-SRP) and anti-3-hydroxy-3-methylglutaryl-coenzyme A reductase (anti-HMGCR) antibodies and prominent necrosis with limited inflammation support the corresponding IMNM subsets. ASyS is associated with antisynthetase antibodies and combinations of myositis, interstitial lung disease, arthritis, and related features; muscle biopsy may show perifascicular necrosis. Finger-flexor and quadriceps weakness, together with endomysial inflammation and rimmed vacuoles, supports sIBM, although sampling can miss characteristic pathology [1,2,8]. MSAs refine clinical classification rather than replacing phenotype assessment [8]. Muscle magnetic resonance imaging (MRI) depicts the distribution of edema and fatty replacement and complements clinical and biopsy findings; overlapping patterns limit its specificity [1,9].

Mitochondria are abundant in skeletal muscle and support the energy demands of muscle contraction [10,11]. Beyond their canonical role in adenosine triphosphate (ATP) generation via oxidative phosphorylation (OXPHOS), particularly in oxidative fiber types, mitochondria regulate reactive oxygen species (ROS) production, apoptotic signaling, calcium homeostasis, and innate immune responses [12,13]. In a mouse model of spontaneous myositis, impaired mitochondrial function can disturb muscle energy metabolism, while antioxidant treatment improved muscle function and preserved mitochondrial structure and respiration, with preventive treatment also reducing muscle inflammation [14]. Mitochondrial DNA (mtDNA), a maternally inherited 16.6 kb double-stranded circular DNA molecule present at hundreds to thousands of copies per cell, encodes 13 OXPHOS complex subunits, 22 transfer RNAs (tRNAs), and 2 ribosomal RNAs (rRNAs), all of which are essential for respiratory chain function [15]. Oxidative stress and impaired mtDNA maintenance can damage mtDNA and compromise mitochondrial gene expression and respiratory function [16,17]. Upon cellular damage or impaired organellar membrane integrity, mtDNA can be released into the cytosol or extracellular milieu, where it acts as a damage-associated molecular pattern (DAMP) and engages pattern-recognition receptors, thereby triggering inflammatory cascades [18,19]. Inflammation can also impair mitochondrial function [14].

Accumulating evidence suggests that mtDNA abnormalities, including large-scale mtDNA deletions, copy number alterations, and increased deletion heteroplasmy, may be involved in IIM-related myofiber injury and degeneration [20,21]. However, the contribution of mtDNA damage appears to vary markedly among disease subtypes. The strongest evidence to date links mtDNA alterations to inclusion body myositis (IBM) and DM, whereas direct molecular characterization in ASyS, IMNM, and other subtypes remains notably sparse [22,23,24]. This disparity raises important questions regarding whether mtDNA instability constitutes a generalized disease-driving mechanism or a modifier limited to distinct disease subsets. Therefore, summarizing the subtype-specific mtDNA signatures may not only refine our understanding of disease heterogeneity in IIMs but also offer a rational basis for future subtype stratification and the development of mechanism-based, individualized therapeutic strategies. Compared with broader reviews of mitochondrial dysfunction in IIMs and IBM [12,21], this review focuses on mtDNA measurements across subtypes and biological compartments, separates clinical associations from functional evidence for DNA-dependent inflammation, and critically assesses biomarker and therapeutic readiness.

This narrative review was updated through targeted searches of Europe PMC, including PubMed-indexed records, last performed on 20 October 2026, without a lower publication-date limit. Search terms covered IIM subtypes, mtDNA, mitophagy, mitochondrial transplantation, DNA sensing, and mitochondrial quality control. Citation tracing supplemented retrieval, and ClinicalTrials.gov was consulted for trial information. Clinical studies were selected for subtype-defined mitochondrial or mtDNA measurements, and experimental studies for interventions testing mitochondrial injury or inflammatory signaling. Non-IIM studies were included when they directly tested relevant mitochondrial or DNA-sensing mechanisms, with model and tissue contexts specified and their findings distinguished from direct evidence in IIMs.

## 2. Association Between mtDNA Abnormalities and IIM Subtypes

### 2.1. Inclusion Body Myositis (IBM) and the PM-Mito Spectrum

Among IIM subtypes, sIBM exhibits the most prominent mitochondrial pathology. Rimmed vacuoles and aggregated misfolded proteins within myofibers are characteristic features of sIBM, frequently accompanied by a marked increase in the proportion of cytochrome c oxidase (COX)-deficient fibers and ragged-red fibers (RRFs), the latter reflecting aberrant subsarcolemmal mitochondrial accumulation. Clonal expansion of mtDNA deletions in individual muscle fibers can contribute to respiratory chain deficiency when the proportion of deleted molecules becomes sufficiently high [25,26]. The breakpoint distribution patterns of these deletions resemble those observed in physiological aging and primary mitochondrial myopathies, suggesting an accelerated accumulation process that may be potentiated by inflammation and age-related stressors [25,26,27]. In parallel with deletion burden, mtDNA copy number is significantly reduced in sIBM muscle, and these combined abnormalities may contribute to mitochondrial dysfunction [28,29]. Moreover, circulating cell-free mtDNA (cf-mtDNA) levels were higher in a small sIBM cohort [28].

Polymyositis with mitochondrial pathology (PM-Mito) shares pathological and molecular features with sIBM, supporting the hypothesis that it represents an early or incomplete IBM phenotype in a subset of patients. Blume et al. identified multiple mtDNA deletions in 9 of 10 patients with inflammatory myopathy characterized by >3% COX-negative fibers and no rimmed vacuoles [30]. Mean age at onset was higher than in 38 other polymyositis (PM) patients (61 versus 52 years), and disease duration ranged from 0.5 to 11.5 years in the study’s clinical table. A repeat biopsy approximately a decade later showed more COX-negative fibers despite reduced inflammation. Kleefeld et al. compared 27 PM-Mito and 27 IBM biopsies in a cross-sectional study, with mean ages at biopsy of 66 and 69 years, respectively [28]. IBM met the 2011 European Neuromuscular Centre (ENMC) criteria, published in 2013; PM-Mito was defined by endomysial inflammation, invasion of non-necrotic fibers, >3% COX-deficient fibers, and absence of vacuoles. Molecular analyses of smaller subsets identified mtDNA deletions and depletion relative to age-matched controls. These findings support shared mitochondrial pathology, but concurrent inflammation at biopsy leaves temporal order unresolved. Longitudinal clinical and molecular assessment must also account for disease duration, chronic injury and sampling, which age matching alone cannot resolve.

### 2.2. Dermatomyositis (DM)

In DM muscle, mtDNA depletion is associated with perifascicular respiratory chain dysfunction. In a study of 12 patients selected for marked perifascicular COX deficiency, affected regions showed reduced mitochondrial transcripts and expression of complex I and IV subunits, whereas the entirely nuclear-encoded complex II was preserved. DNA analyses in four cases demonstrated reduced mtDNA copy number without an increase in large-scale rearrangements [20]. At the transcriptional level, spatial analysis of treatment-naïve juvenile dermatomyositis (JDM) muscle identified downregulated mitochondrial pathways, supported by an independent bulk RNA-sequencing cohort that also showed reduced mtDNA-encoded transcripts. In the same study, mitochondrial histopathology did not correlate with expression of the interferon (IFN) marker myxovirus resistance protein A (MxA) in 19 untreated JDM patients. This small cross-sectional comparison, however, does not establish that mitochondrial dysfunction is independent of IFN activity [31].

Evidence for DNA-dependent inflammatory signaling comes from studies of serum and circulating immune cells. In anti-nuclear matrix protein 2 (anti-NXP2) antibody-positive DM, serum levels of both cf-mtDNA and cell-free nuclear DNA were elevated, and activation of cyclic GMP–AMP synthase (cGAS)–stimulator of interferon genes (STING) signaling in muscle was associated with perifascicular atrophy. Deoxyribonuclease I (DNase I) pretreatment of patient serum or STING inhibition reduced serum-induced STING phosphorylation in C2C12 myoblasts, supporting DNA-dependent signaling without resolving the relative contributions of mitochondrial and nuclear DNA [32]. In JDM, mitochondrial gene dysregulation correlated with an IFN signature in pretreatment cluster of differentiation 14 (CD14)-positive monocytes, whereas increased oxidized mtDNA was demonstrated in monocytes from patients receiving treatment. The inflammatory activity of oxidized mtDNA was tested separately in healthy-control peripheral blood mononuclear cells (PBMCs), using human mtDNA amplified by polymerase chain reaction (PCR), oxidized in vitro, and delivered with LL37. Oxidized mtDNA increased MX dynamin-like GTPase 1 (MX1) expression, whereas non-oxidized mtDNA did not, and Toll-like receptor 9 (TLR9) antagonism reduced this response [33]. Blood displacement-loop (D-loop) polymorphisms have also been associated with disease in a mixed PM/DM cohort [34]. The study included 10 PM and 76 DM patients classified using the Bohan and Peter criteria, together with 110 healthy controls. These criteria predate modern MSA-based classification; some historically classified PM cases may now be classified as IMNM, ASyS, or IBM [1,2,8]. These observations span distinct tissues, cell populations and experimental systems; their mechanistic relationship in patients remains unresolved.

### 2.3. Antisynthetase Syndrome (ASyS), Immune-Mediated Necrotizing Myopathy (IMNM), and Other Subtypes

Mitochondrial abnormalities have also been documented in ASyS and selected IMNM subgroups [22,35]. In ASyS, electron microscopy has identified abnormal mitochondrial morphology and cristae, together with ultrastructural signs of mitophagy [22]. Sera from patients with ASyS and DM impair mitochondrial respiration and contractile function in isolated mouse muscle preparations [24]; these experiments did not establish mtDNA as the mediator [24].

In anti-SRP-positive and anti-HMGCR-positive IMNM, experimental studies support a pathogenic role for autoantibodies and complement in myofiber injury [36,37]. An observational anti-HMGCR study also found more COX-negative/succinate dehydrogenase (SDH)-positive fibers than in age-matched controls [35].

Antimitochondrial antibody (AMA)-associated IIMs provide evidence from both muscle pathology and circulating mtDNA measurements. In one cohort, patients were grouped by ENMC-based IIM diagnosis and AMA positivity, encompassing different muscle pathological patterns [38]. AMAs recognize mitochondrial protein antigens. Among 37 biopsies, 30 showed an IMNM-like pattern; COX deficiency or reduced activity occurred in 12 of 36 evaluable biopsies, and RRFs in 2 of 37. In the serum subset of 21 patients, cf-mtDNA levels were higher than in healthy controls but did not differ significantly from AMA-negative IMNM, DM, or ASyS disease controls [38].

Together, these findings support paired assessment of muscle mtDNA integrity and copy number, mitochondrial function, and circulating mtDNA within defined IIM subtypes (Table 1). Histopathology and circulating mtDNA abundance alone cannot establish a specific muscle mtDNA abnormality or its causal contribution to injury.

## 3. Core Types and Molecular Characteristics of mtDNA Abnormalities in IIMs

### 3.1. Large-Scale mtDNA Deletions

Large-scale mtDNA deletions have been reported in muscle from patients with sIBM and PM-Mito [25,28,39]. Interpreting these findings requires distinguishing the proportion of deletion-positive samples or fibers, the number of distinct deletion species, and the abundance of deleted mtDNA molecules. For a specified deletion, heteroplasmy is the fraction of mtDNA molecules carrying that deletion in the analyzed material [26,40]. In sIBM, single-fiber analysis has identified high deletion levels in some COX-deficient fibers, whereas corresponding muscle homogenates yielded much lower estimates [26]. Whole-muscle measurements therefore cannot resolve how deletions are distributed among individual fibers. Long-range PCR can detect mtDNA deletions but cannot, by itself, quantify deletion burden [41].

### 3.2. mtDNA Copy Number Alterations and Depletion

Decreased mtDNA copy numbers have been reported in IBM and PM-Mito muscle and in perifascicular DM regions [20,28,42]. The copy number must be interpreted with its denominator and sampling method. For example, relative quantitative PCR (qPCR) used mitochondrial NADH dehydrogenase subunit 1 (MT-ND1) normalized to nuclear β2-microglobulin (B2M) in IBM/PM-Mito muscle [28], whereas the blood PM/DM study normalized MT-ND1 to human β-hemoglobin (HGB) and reported increased relative abundance [34]. Whole-muscle estimates may be affected by muscle-fiber loss and inflammatory-cell admixture [42]; the blood study did not report adjustment for blood-cell composition [34]. Region-specific measurements therefore address different questions from whole-muscle measurements [20]. The copy number estimates mtDNA abundance, not mitochondrial mass, respiratory capacity, or the proportion of pathogenic genomes [20,42]. Differences in tissue source, patient groups, and normalization limit direct comparison of blood increases with muscle depletion.

### 3.3. Somatic mtDNA Variants and D-Loop Polymorphisms

Deep sequencing also suggested an increase in somatic mtDNA single-nucleotide variants in IBM muscle compared with age-matched controls [40]. An enrichment of the mtDNA D-loop allele 16304C has been reported in blood samples from patients with DM [34]. In the combined PM/DM cohort, the 16304C allele was associated with lower interleukin-4 (IL-4) levels, and the 16519C allele was associated with antinuclear antibody (ANA) positivity [34]. These blood associations concern sequence polymorphisms, not demonstrated acquired pathogenic mutations; inheritance was not directly tested. The study did not report ancestry or mitochondrial haplogroup adjustment, leaving population structure as a potential confounder [34].

### 3.4. Oxidative Modification, Conformation and Release of mtDNA

Under oxidative stress and impaired mitochondrial quality control, mtDNA can undergo oxidative base modification [43]. In JDM patients receiving treatment, CD14^+^ monocytes contain increased oxidized mtDNA (ox-mtDNA). Experimentally oxidized mtDNA induced greater expression of the interferon-stimulated gene (ISG) MX1 than unoxidized mtDNA in healthy-donor PBMCs under LL37-assisted uptake conditions, supporting sufficiency for ISG induction in that system [33]. In addition, biochemical experiments demonstrated binding of NOD-like receptor family pyrin domain-containing 3 (NLRP3) to oxidized mtDNA fragments [44]. Release of mtDNA has also been linked to cGAS–STING activation in myofibers from zinc metallopeptidase STE24 (*Zmpste24*)-deficient progeria mice [45]. Ultraviolet B (UVB)-induced mitochondrial oxidative stress promotes cytosolic accumulation of mtDNA in the Z conformation in keratinocytes, where IFN-induced Z-DNA-binding protein 1 (ZBP1) stabilizes Z-DNA and amplifies cGAS–STING signaling. In this model, mtDNA depletion reduced UVB-induced IFN responses, while DNase I, but not ribonuclease A (RNase A), reduced Z-DNA staining [46]. ZBP1 expression was also increased in skin samples from patients with adult DM and JDM [46]. These findings concern keratinocytes and skin; this study did not examine the same mechanism in affected muscle.

### 3.5. Mitochondrial Quality Control and mtDNA Homeostasis

Studies in non-IIM models link mitochondrial quality control to mtDNA homeostasis and inflammatory signaling. Stabilization of PTEN-induced kinase 1 (PINK1) on depolarized mitochondria recruits Parkin and promotes mitophagy [47]. Depletion of the mitophagy receptor BCL2-interacting protein 3 (BNIP3) impaired mitochondrial and lysosomal homeostasis and promoted mtDNA-associated, TLR9-dependent inflammation in C2C12 myotubes; it also exacerbated muscle inflammation in aged mice [48]. BCL2-interacting protein 3-like (NIX/BNIP3L) and FUN14 domain-containing 1 (FUNDC1) were identified as mitophagy receptors in erythroid and hypoxia models, respectively [49,50]. Mitochondrial Lon peptidase 1 (LONP1) depletion impaired mitochondrial protein maturation and mtDNA maintenance in fibroblasts [51]. Loss of caseinolytic mitochondrial matrix peptidase proteolytic subunit (CLPP), the proteolytic component of ClpXP, induced mtDNA-dependent cGAS–STING signaling in mouse embryonic fibroblasts, with mtDNA depletion reducing this response [52].

Mitochondrial dynamics also matter: dynamin-related protein 1 (DRP1)-mediated fission and fusion mediated by mitofusins 1 and 2 (MFN1/2) or optic atrophy 1 (OPA1) must remain coordinated. In non-IIM muscle models, disrupting either fission or fusion promoted inflammatory signaling, with mtDNA trafficking to endosomal compartments implicated in TLR9 activation [53]. This provides an argument against treating DRP1 inhibition as universally protective. Moreover, mdivi-1 has DRP1-independent effects on complex I, complicating attribution of its effects to fission inhibition [54]. In sIBM/PM-Mito, COX-deficient fibers and mitochondrial accumulation indicate organellar pathology [23,28], while increased phosphorylated Ser65 ubiquitin, a mitophagy-associated marker, has been demonstrated in IBM muscle [55]. These static observations do not distinguish increased mitophagy initiation from impaired downstream clearance. Therapeutic modulation requires functional validation within each IIM subtype.

## 4. Core Mechanisms Linking mtDNA Abnormalities to Myositis Pathogenesis

### 4.1. cGAS–STING Pathway and Type I Interferon Response

The cGAS–STING pathway mediates cytosolic DNA sensing and type I IFN induction [56]. In experimental models outside IIMs, mtDNA stress induced by mitochondrial transcription factor A (TFAM) deficiency promotes mtDNA release into the cytosol and cGAS-dependent signaling [18]. DNA binding activates cGAS to synthesize cyclic GMP–AMP (cGAMP), which subsequently activates the STING–TANK-binding kinase 1 (TBK1)–interferon regulatory factor 3 (IRF3) axis, inducing type I IFN production and expression of a broad repertoire of ISGs [18,56].

In IIMs, STING phosphorylation supports pathway activation in IBM/PM-Mito muscle [28]. In anti-NXP2-positive DM, cytosolic double-stranded DNA colocalized with cGAS in muscle, and increased pathway phosphorylation was detected in patients with perifascicular atrophy (PFA). DNase I treatment of sera from patients with PFA and STING inhibition in serum-exposed C2C12 myoblasts support DNA-associated signaling [32]. IFN-β also impairs mitochondrial respiration in human myotubes, an effect prevented by the ROS scavenger N-acetylcysteine, supporting one possible reverse link from inflammation to mitochondrial injury [57]. Plasma-derived extracellular vesicles from patients with DM carry both mitochondrial and nuclear DNA. Nuclease treatment of vesicle preparations, with or without Triton X-100-mediated membrane disruption, and STING knockdown or inhibition in PBMCs reduced inflammatory responses, supporting a contribution from vesicle-associated DNA and STING involvement [58]. The mtDNA-specific contribution remains unresolved across these patient and experimental observations.

Patients with JDM showed calcified mitochondria in muscle and elevated plasma mtDNA, with higher mtDNA levels in those with calcinosis [59]. In the same study, experiments using the RH30 rhabdomyosarcoma cell line showed that calcium-phosphate exposure induced mitochondrial dysfunction and ISG expression; IFN-α enhanced mitochondrial ROS-dependent calcification, and the STING inhibitor H-151 reduced interferon-stimulated gene 15 (ISG15) expression and calcification. Isolated-mitochondria experiments separately demonstrated mtDNA release. These experimental findings require validation in primary human skeletal muscle cells and patient muscle tissue [59].

### 4.2. The TLR9 Signaling Pathway

TLR9 functions as an endosomal pattern-recognition receptor that recognizes unmethylated cytosine–phosphate–guanine (CpG) motifs in DNA, including mtDNA. Consequently, mtDNA that is released into the extracellular space and subsequently internalized into endosomes can activate TLR9. This downstream signaling proceeds via the myeloid differentiation primary response 88 (MyD88)-dependent pathway, leading to nuclear factor-kappa B (NF-κB) activation and the production of pro-inflammatory cytokines, including tumor necrosis factor-α (TNF-α) and interleukin-6 (IL-6) [19,60]. TLR9 can also signal through MyD88 and interferon regulatory factor 7 (IRF7) to induce type I IFN, as demonstrated in plasmacytoid dendritic cells [61]. This cell-dependent branch is distinct from NF-κB-driven cytokine transcription.

In pretreatment JDM PBMCs, the TLR9 antagonist A151 reduced MX1 and radical S-adenosylmethionine domain-containing 2 (RSAD2) expression, whereas the cGAS inhibitor G140 reduced MX1 without a significant effect on RSAD2 [33]. In separate experiments using healthy-control PBMCs, A151, but not G140, significantly reduced ox-mtDNA-induced MX1 expression. LL37-assisted uptake may influence access to sensing compartments [33]. Mixed PBMC responses do not identify the responding cell type or demonstrate the MyD88–IRF7 branch in patient monocytes. In non-IIM experimental muscle models, disrupted mitochondrial dynamics and BNIP3 deficiency can promote mtDNA mislocation and TLR9-dependent inflammatory signaling [48,53]. Establishing endogenous mtDNA dependence and pathway specificity in IIMs requires orthogonal perturbations and controls for viability and inhibitor specificity.

### 4.3. NLRP3 Inflammasome Activation

The NLRP3 inflammasome is a cytosolic multiprotein complex that activates caspase-1 to process pro-interleukin-1β (pro-IL-1β) and pro-interleukin-18 (pro-IL-18) into mature cytokines [62]. Priming increases NLRP3 and pro-IL-1β expression, whereas activation drives inflammasome assembly [62,63].

In IBM muscle, increased NLRP3 and apoptosis-associated speck-like protein containing a caspase recruitment domain (ASC) expression accompanies altered mitophagy markers [55]. A role for mtDNA in this pathway is supported by experiments outside IIMs: cytosolic ox-mtDNA promoted NLRP3 assembly and caspase-1 activation in primed mouse macrophages [62,63], while biochemical assays demonstrated direct mtDNA–NLRP3 binding [44]. Studies of nucleus pulposus cells and brown adipose tissue further associate cytosolic mtDNA accumulation with NLRP3 signaling in models of disk degeneration and thermogenic dysfunction, respectively [64,65]. These findings support mtDNA as a potential mediator linking mitochondrial injury to NLRP3 activation, but do not establish that endogenous mtDNA initiates or sustains this response in human IIM muscle.

**Figure 1:**
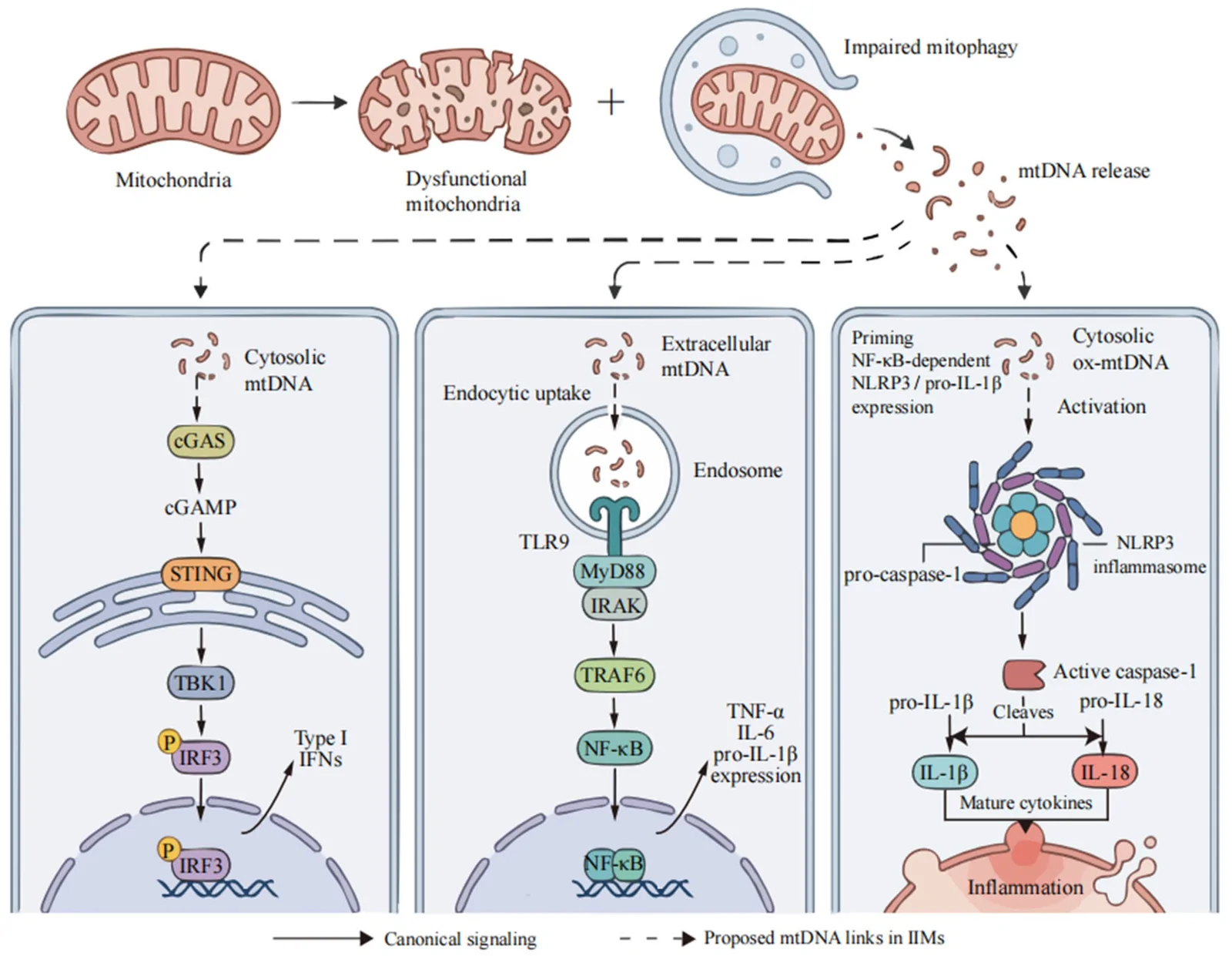
Mitochondrial DNA (mtDNA)-associated innate immune pathways relevant to idiopathic inflammatory myopathies (IIMs). Solid arrows indicate canonical signaling; dashed arrows indicate proposed mtDNA-related links in IIMs. Evidence includes ex vivo cGAS/TLR9 inhibitor responses in juvenile dermatomyositis peripheral blood mononuclear cells and increased NLRP3/ASC expression in inclusion body myositis muscle; the complete endogenous mtDNA-driven cascades remain unconfirmed in IIMs. TLR9 can also induce type I interferons through MyD88–IRF7 in plasmacytoid dendritic cells; this branch is not shown. ASC, apoptosis-associated speck-like protein containing a caspase activation and recruitment domain; cGAMP, cyclic GMP–AMP; cGAS, cyclic GMP–AMP synthase; IFNs, interferons; IL, interleukin; IRAK, interleukin-1 receptor-associated kinase; IRF3, interferon regulatory factor 3; IRF7, interferon regulatory factor 7; MyD88, myeloid differentiation primary response 88; NF-κB, nuclear factor-kappa B; NLRP3, NOD-like receptor family pyrin domain-containing 3; ox-mtDNA, oxidized mtDNA; P, phosphorylation; STING, stimulator of interferon genes; TBK1, TANK-binding kinase 1; TLR9, Toll-like receptor 9; TNF-α, tumor necrosis factor-α; TRAF6, tumor necrosis factor receptor-associated factor 6.

Taken together, DNA sensing through cGAS–STING and TLR9, together with mtDNA-associated NLRP3 activation, may contribute to inflammatory amplification in IIMs: IFN-driven mitochondrial dysfunction and mtDNA release could reinforce inflammation [57,58,59]. This integrated model combines evidence from IIMs and other experimental systems; a complete feedback loop has not been established in any IIM subtype (Figure 1).

## 5. Biomarkers of mtDNA Abnormalities in IIMs

### 5.1. Circulating Cell-Free Mitochondrial DNA (Cf-mtDNA)

cf-mtDNA is a candidate circulating biomarker in IIMs, although its clinical associations vary across cohorts. In sIBM, relative qPCR for MT-ND1 and mitochondrial NADH dehydrogenase subunit 4 (MT-ND4) detected higher serum levels in nine patients than in seven healthy controls after exclusion of two control outliers [28]. Similarly, absolute serum MT-ND1 qPCR using cloned standards in 21 AMA-associated IIM patients showed higher levels than in 23 healthy controls, but no significant difference from 66 disease controls and no significant correlations with the clinical and pathological measures assessed. Six patients were receiving treatment at sampling [38].

Associations with disease activity and calcinosis have been reported in DM. Serum MT-ND1 qPCR in 20 treatment-naïve anti-NXP2-positive DM patients showed higher cf-mtDNA levels than in 37 anti-NXP2-negative DM patients, 37 IMNM patients, and 25 healthy controls. Levels correlated with Myositis Disease Activity Assessment Visual Analogue Scales (MYOACT) total and cutaneous scores (ρ = 0.458 and 0.529, respectively) [32]. A JDM study using qPCR to measure plasma mtDNA included 19 patients with calcinosis, 42 without calcinosis, and 17 healthy controls, and found higher levels in patients with calcinosis than in those without [59].

Clinical translation of cf-mtDNA requires assay standardization and validation of added clinical value. Changes in cf-mtDNA are not confined to IIMs and have also been reported after kidney transplantation and in patients with breast tumors [66,67]. Platelet activation, centrifugation and freezing during plasma preparation can affect measured abundance [68], while IIM studies also differ in DNA extraction and quantification procedures [28,38]. Serum and plasma measurements should therefore not be pooled without protocol harmonization. qPCR of total extracted DNA alone does not distinguish free mtDNA from mtDNA associated with vesicles or retained within circulating mitochondria, or establish its cellular source or inflammatory activity [58,68]. Prospective longitudinal studies with standardized processing should test whether cf-mtDNA adds clinical value beyond conventional disease assessment after accounting for subtype, treatment and other tissue injury.

### 5.2. Tissue-Based Markers: mtDNA Copy Number and Histopathological Indicators

Muscle mtDNA analysis complements histopathology by linking molecular abnormalities to affected fibers and regions. In sIBM, deletion load varies among individual muscle fibers, whereas selected DM biopsies show lower mtDNA abundance in COX-deficient than in COX-positive regions [20,26,41]. COX/SDH histochemistry identifies respiratory-deficient fibers, while long-range PCR and sequencing characterize deletions and rearrangements; validated quantitative assays are required to measure copy number and deletion heteroplasmy [20,25,41].

Biopsy interpretation should specify the sampled muscle, disease stage, and regions selected for analysis [9,20,26]. COX deficiency can also be segmental along individual fibers [25]. The potential clinical relevance of mitochondrial pathology is illustrated by a retrospective multicenter study comparing 25 patients with age-exceeding COX-negative fibers to 20 myositis controls without significant mitochondrial abnormalities [69]. Patients met the 2017 European League Against Rheumatism/American College of Rheumatology (EULAR/ACR) IIM classification criteria, and definite IBM under the study’s 2013 criteria was excluded. Mitochondrial pathology was associated with treatment refractoriness and worse outcomes, although the groups differed in subtype composition, baseline severity, and time from symptom onset to biopsy. Four patients were subsequently classified as clinically defined IBM; the authors noted that all nine PM-Mito patients would already have met the 2024 ENMC criteria for confirmed IBM at baseline [69]. Although mtDNA was not assessed, these findings motivate longitudinal testing of its prognostic value beyond histopathology, using consistent subtype classification and accounting for baseline severity, disease duration, treatment exposure and diagnostic reclassification.

## 6. Therapeutic Strategies Targeting Mitochondrial Injury and Downstream Inflammation

### 6.1. Antioxidant Intervention and Clearance of Mitochondrial ROS

Antioxidant interventions aim to limit mitochondrial oxidative injury and downstream inflammation. N-acetylcysteine (NAC) is a thiol antioxidant and glutathione precursor. NAC suppressed ox-mtDNA-induced MX1 expression in healthy-control PBMCs and reduced MX1 and RSAD2 expression in treatment-naïve JDM PBMCs [33]. In inducible T-cell costimulator (*Icos*)-deficient non-obese diabetic (NOD) mice with spontaneous autoimmune myositis, NAC improved muscle function and preserved mitochondrial structure and respiration [14]. Methyl 3,4-dihydroxybenzoate (MDHB) reduced oxidative stress, inflammation, and necroptosis-associated signaling in experimental autoimmune myositis, and inhibited pyroptosis in cultured macrophages [70]. In the JDM-related RH30 model, MitoTEMPO reduced IFN-α-associated mitochondrial ROS and calcification [59]. Outside IIMs, 21 days of MitoQ supplementation attenuated exercise-induced mtDNA damage in healthy men: 24 were randomized, with muscle biopsies from 10 participants, five per group. The muscle endpoint was lesion frequency measured by long-amplicon quantitative PCR [71]. MitoTEMPO and SS-31 also improved vascular or cerebrovascular function in aging models [72,73]. Thus, MitoQ provided a direct mtDNA damage endpoint outside IIMs; anti-inflammatory, respiratory, and functional benefits in the other models do not by themselves establish repair of mtDNA lesions.

Antioxidant treatment should also account for dose and site of action because ROS participate in physiological adaptation. In healthy men, vitamins C and E attenuated selected exercise-induced metabolic adaptations [74], with uncertain relevance to NAC or mitochondria-targeted compounds in IIMs. Long-term studies should assess oxidative injury alongside muscle function.

### 6.2. Targeting Nucleic Acid-Sensing Pathways and Downstream IFN Signaling

The cGAS–STING and TLR9 pathways are investigational targets for DNA-associated inflammation in selected IIM settings; neither pathway is specific to mtDNA. In ex vivo experiments using PBMCs from treatment-naïve patients with JDM, the TLR9 antagonist A151 reduced MX1 and RSAD2 expression, whereas the cGAS inhibitor G140 reduced MX1 without a significant effect on RSAD2 [33]. In addition, Janus kinase (JAK) inhibitors can act further downstream by blocking cytokine signaling. Ruxolitinib and baricitinib prevented mitochondrial ultrastructural abnormalities in human myotubes exposed to type II IFN (IFN-γ) in vitro [14]. This type II IFN model differs from the type I IFN responses discussed above. Earlier clinical evidence largely comprised uncontrolled studies [75]. More recently, a phase III randomized controlled trial (RCT) involving 241 adults with DM demonstrated the efficacy of brepocitinib 30 mg daily at 52 weeks, whereas the 15 mg dose did not meet the primary efficacy endpoint. Patients were randomized 1:1:1; the mean Total Improvement Score (TIS) was 46.5 with 30 mg, 37.5 with 15 mg, and 31.2 with placebo, a difference of 15.3 (95% confidence interval 6.7 to 24.0) for 30 mg and 6.3 (95% confidence interval −2.4 to 14.9) for 15 mg. Serious infections occurred in 10% of the 30 mg group versus 1% of the placebo group [76]. This trial supports clinical benefit from downstream tyrosine kinase 2 (TYK2)/JAK1 inhibition in DM. Because these inhibitors affect multiple cytokine pathways, including both IFN families, clinical efficacy does not establish an mtDNA-specific mechanism.

In experimental models, cGAS–STING supports antiviral responses and antitumor T-cell priming [18,77], suggesting that systemic inhibition could impair protective immunity; these studies do not quantify clinical risk in IIMs. Tissue-directed delivery warrants investigation, and trials should account for IIM subtype, infection and malignancy risk, and concomitant immunosuppression. mtDNA and IFN measures require validation before guiding patient selection for cGAS–STING inhibition.

### 6.3. Mitochondrial Transplantation

Mitochondrial transplantation aims to restore cellular bioenergetics by delivering functional exogenous mitochondria [78]. PN-101, an allogeneic mitochondrial preparation derived from human umbilical cord mesenchymal stem cells, improved mitochondrial respiration and myogenic differentiation in patient-derived myoblasts in vitro and reduced muscle inflammation in C protein-induced myositis mice [79].

In an open-label phase I/IIa trial (NCT04976140; accessed on 20 October 2026), nine patients with refractory PM/DM received a single intravenous infusion of PN-101. Improvements in mean International Myositis Assessment and Clinical Studies Group Total Improvement Score (IMACS-TIS) were reported at week 12, with no severe adverse drug reactions during follow-up. Efficacy was reported for eight patients; the main report did not clearly explain the difference from the nine treated patients. Background glucocorticoid and immunosuppressant doses were required to remain stable, and intravenous immunoglobulin was prohibited. The uncontrolled design, small sample and short follow-up preclude firm conclusions about efficacy, dose–response or long-term safety [79]. A randomized, double-blind, placebo-controlled phase II trial has subsequently been registered on ClinicalTrials.gov (NCT07122648; accessed on 20 October 2026).

Translation requires assessment of mitochondrial function and mtDNA integrity in both autologous and allogeneic preparations, including potentially pathogenic variants [78]. Extracellular mitochondrial preparations promoted immune activation in a murine cardiac allograft model [80], but this does not establish PN-101 immunogenicity. In an endothelial-cell model, mitochondrial transplantation activated PINK1–Parkin-dependent mitophagy and increased mitochondrial biogenesis with negligible fusion into the recipient network [81]. In dystrophin-deficient *mdx* mice, intramuscular injection of mitochondria isolated from healthy mice reduced calcium deposition in skeletal muscle and attenuated calcium overload in isolated quadriceps mitochondria [82]. Separately, PN-101 restored intracellular free calcium levels towards baseline in lipopolysaccharide (LPS)-stimulated THP-1-derived macrophages in vitro [83]. These studies neither examined IIMs nor directly demonstrated calcium buffering by donor mitochondria, leaving it unclear whether remodeling, integration, or calcium handling contributes to PN-101 activity in this setting. Studies comparing systemic and local delivery should assess biodistribution and dose–response, while donor mtDNA tracking should be linked to direct measurements of mitochondrial fate and function. Longer controlled trials with immune monitoring are needed to establish clinical benefit and safety.

### 6.4. Exercise Intervention as a Non-Pharmacological Modulator of Mitochondrial Function

Regular exercise training constitutes an important non-pharmacological component in IIM management, with emerging evidence of associated mitochondrial adaptations. Endurance exercise can upregulate peroxisome proliferator-activated receptor gamma coactivator 1-alpha (PGC-1α) and other key regulators of mitochondrial biogenesis via multiple signaling pathways, thereby promoting mitochondrial biogenesis and improving aerobic metabolism in skeletal muscles [84]. In a chloroquine-induced rat model used to study sIBM-like pathology, resistance training improved mitochondrial quality-control markers and reduced β-amyloid accumulation [85]. Chloroquine-induced lysosomal dysfunction models selected features of vacuolar myopathy, with uncertain relevance to the immune pathogenesis of human sIBM. In experimental autoimmune myositis mice, high-intensity interval training (HIIT) delivered by electrical stimulation of the plantar flexor muscles increased complex I, III, and IV protein expression, citrate synthase and COX activity, and muscle fatigue resistance [86]. A multicenter RCT in 23 patients with recent-onset IIMs (4 PM, 10 DM, and 9 ASyS) with 20 completing follow-up found that 12 weeks of HIIT improved aerobic capacity more than moderate exercise, with stable disease activity. HIIT was supervised three times weekly initially, then one to three times weekly; mitochondrial protein analyses included 13 patients (7 HIIT and 6 controls) [87]. These findings cannot be generalized to sIBM, IMNM, or all disease stages.

Together, these findings support structured, individually adapted exercise to improve physical capacity in the studied IIM populations. Exercise tolerance, disease activity, and cardiopulmonary involvement should guide supervision and progression; effects on mtDNA integrity remain to be tested (Table 2).

## 7. Conclusions

**Figure 2:**
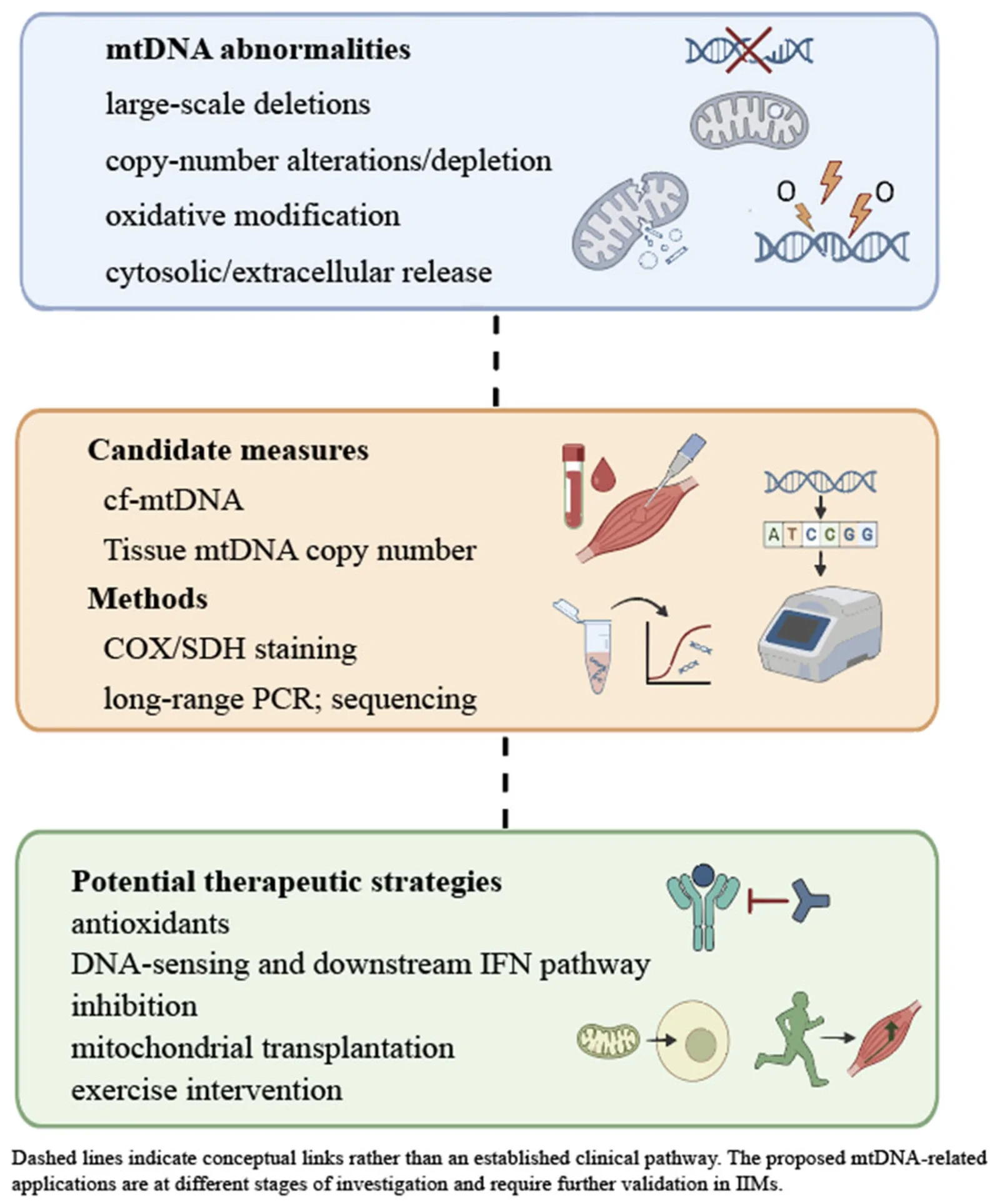
mtDNA-related abnormalities, candidate biomarkers, assessment methods and potential therapeutic strategies in IIMs. Evidence for the therapeutic strategies and their mtDNA-related endpoints is summarized in Table 2. mtDNA, mitochondrial DNA; IIMs, idiopathic inflammatory myopathies; cf-mtDNA, cell-free mitochondrial DNA; COX, cytochrome c oxidase; SDH, succinate dehydrogenase; PCR, polymerase chain reaction; IFN, interferon.

The strongest evidence for mtDNA abnormalities in IIMs concerns structural and quantitative changes in IBM/PM-Mito muscle and regional depletion in selected DM biopsies. Findings in DM and JDM extend the evidence to circulating DNA and monocyte ox-mtDNA, while skin models provide additional mechanistic insight into mtDNA sensing. Experiments in IIM-related and non-IIM systems support DNA-dependent inflammatory signaling and inflammation-induced mitochondrial dysfunction. Together, these findings provide a basis for investigating mtDNA-related biomarkers and therapeutic strategies (Figure 2), while the mtDNA-specific contribution to inflammation in patients remains the central mechanistic question.

Priorities include longitudinal subtype-defined cohorts, standardized cf-mtDNA sampling and assays, and paired assessment of inflammation and mitochondrial function. Mechanistic studies should combine selective perturbation and rescue with controls for nuclear DNA, respiration, and viability, because mtDNA depletion itself can disrupt OXPHOS. Longitudinal studies should test whether mtDNA measurements improve disease monitoring and prediction of treatment response beyond conventional clinical and inflammatory assessments.

## Figures and Tables

**Table 1:** Subtype-specific mtDNA abnormalities and associated pathological features across IIM subtypes.

Subtype/Group	mtDNA Findings and Material	Key Pathology	Study Type and Interpretation
sIBM	Muscle: deletions, copy number ↓, deletion heteroplasmy ↑ [25,28,29]; serum: cf-mtDNA ↑ [28]	COX-deficient and ragged-red fibers	Observational; whether mtDNA injury precedes inflammation remains unresolved
DM	Muscle: perifascicular depletion [20]; serum: cf-mtDNA ↑ in anti-NXP2 DM [32]; blood: copy number ↑ and D-loop SNP associations [34]; treated JDM CD14^+^ monocytes: ox-mtDNA ↑ [33]	Perifascicular respiratory chain dysfunction [20]	Patient observations and C2C12/PBMC experiments; causal links between compartments unproven
PM	Blood: relative copy number ↑; no significant increase in D-loop SNP count [34]	Muscle mtDNA not assessed	Case–control, *n* = 10; Bohan and Peter criteria [34]
PM-Mito	Muscle: multiple deletions [28,30]; copy number ↓ [28]	IBM-like mitochondrial pathology	Cross-sectional cohort, 27 PM-Mito/27 IBM [28]; 10-case series with limited follow-up [30]. Early IBM stage remains hypothetical
ASyS	mtDNA lesions poorly characterized	Abnormal mitochondria/cristae; ultrastructural signs of mitophagy [22]	Muscle ultrastructure and serum-exposed isolated mouse muscle [22,24]; mtDNA mediation unproven
IMNM	mtDNA alterations poorly characterized	Necrosis/regeneration; excess COX-negative/SDH-positive fibers in anti-HMGCR IMNM [35,36,37]	Biopsy and antibody-transfer studies in anti-SRP/anti-HMGCR subsets [36,37]
AMA-positive IIM	Serum: cf-mtDNA ↑ versus healthy controls, but no significant difference versus AMA-negative IIM controls; muscle mtDNA uncharacterized [38]	Predominantly IMNM-like; COX abnormalities; rare ragged-red fibers [38]	Observational cohort; ENMC-based IIM diagnosis plus AMA positivity; serum *n* = 21 [38]

Notes: ↑, increased; ↓, decreased; *n*, sample size. IIM, idiopathic inflammatory myopathy; sIBM, sporadic inclusion body myositis; IBM, inclusion body myositis; DM, dermatomyositis; JDM, juvenile dermatomyositis; PM, polymyositis; PM-Mito, polymyositis with mitochondrial pathology; ASyS, antisynthetase syndrome; IMNM, immune-mediated necrotizing myopathy; AMA, antimitochondrial antibodies; mtDNA, mitochondrial DNA; cf-mtDNA, cell-free mitochondrial DNA; ox-mtDNA, oxidized mitochondrial DNA; D-loop, displacement loop; SNP, single-nucleotide polymorphism; COX, cytochrome c oxidase; SDH, succinate dehydrogenase; PBMC, peripheral blood mononuclear cell; CD14, cluster of differentiation 14; NXP2, nuclear matrix protein 2; SRP, signal recognition particle; HMGCR, 3-hydroxy-3-methylglutaryl-coenzyme A reductase; ENMC, European Neuromuscular Centre.

**Table 2:** Summary of mitochondrial and downstream anti-inflammatory therapeutic strategies and their translational status in IIMs.

Intervention/Target	Study Population/Evidence Stage	Key Findings
NAC—antioxidant	JDM PBMCs, ex vivo; mouse myositis	↓ ISG expression in PBMCs; improved muscle function and respiration in mice [14,33]
MDHB—antioxidant	EAM mice; cell models	↓ Oxidative stress and necroptosis markers; ↓ macrophage pyroptosis in vitro [70]
MitoQ—mitochondrial antioxidant	RCT in healthy men; biopsy subset *n* = 10	21-day supplementation reduced exercise-induced muscle mtDNA lesion frequency assessed by LA-qPCR [71]
MitoTEMPO—mitochondrial ROS scavenging	JDM-related RH30 model; aged mice	↓ IFN-α-associated ROS/calcification [59]; improved vascular function [72]
SS-31—cardiolipin interaction	Non-IIM aging models	Improved mitochondrial and cerebrovascular function [73]
Ruxolitinib/baricitinib—JAK1/JAK2	Human myotubes, in vitro	Prevented IFN-γ-induced mitochondrial ultrastructural abnormalities [14]
Brepocitinib—TYK2/JAK1	Adult DM phase III RCT, *n* = 241	30 mg/day improved week-52 TIS versus placebo; 15 mg/day missed the primary endpoint. Serious infections: 10% versus 1% with 30 mg versus placebo [76]
A151—TLR9; G140—cGAS	Treatment-naïve JDM PBMCs, ex vivo	A151: ↓ MX1/RSAD2; G140: ↓ MX1, RSAD2 NS [33]; endogenous mtDNA dependence unproven
PN-101—allogeneic hUC-MSC mitochondria	Refractory PM/DM; uncontrolled phase I/IIa, *n* = 9; single intravenous infusion. Phase II registered (NCT07122648)	Week-12 TIS improvement; efficacy figure *n* = 8; infusion reaction [79]. Long-term donor persistence/integration unresolved
Exercise—HIIT/resistance training	Recent-onset PM/DM/ASyS RCT, *n* = 23; CQ-induced rat myopathy and EAM mice [85,86]	Supervised HIIT improved aerobic capacity versus standard exercise; mitochondrial protein analyses, *n* = 13 [87]

Notes: ↓, decreased; NS, not statistically significant; *n*, sample size; IIM, idiopathic inflammatory myopathy; DM, dermatomyositis; JDM, juvenile dermatomyositis; PM, polymyositis; ASyS, antisynthetase syndrome; EAM, experimental autoimmune myositis; mtDNA, mitochondrial DNA; NAC, N-acetylcysteine; MDHB, methyl 3,4-dihydroxybenzoate; PBMC, peripheral blood mononuclear cell; ISG, interferon-stimulated gene; ROS, reactive oxygen species; IFN, interferon; JAK, Janus kinase; TYK2, tyrosine kinase 2; TLR9, Toll-like receptor 9; cGAS, cyclic GMP–AMP synthase; hUC-MSC, human umbilical cord mesenchymal stem cell; TIS, Total Improvement Score; RCT, randomized controlled trial; HIIT, high-intensity interval training; CQ, chloroquine; LA-qPCR, long-amplicon quantitative polymerase chain reaction; MX1, MX dynamin-like GTPase 1; RSAD2, radical S-adenosylmethionine domain-containing 2. RH30 is a human rhabdomyosarcoma cell line.

## Data Availability

No new data were created or analyzed in this study. Data sharing is not applicable to this article.
